# Perioperative care of patients with chronic myeloid leukemia on tyrosine kinase inhibitors

**DOI:** 10.3389/fonc.2026.1899207

**Published:** 2026-09-25

**Authors:** Mutasim Binidris, Daniela Žáčková, Rasha Kaddoura, Rola Ghasoub, Ahmet Emre Eşkazan, Giuseppe Saglio, Mohamed Yassin

**Affiliations:** 1 Department of Medical Education, Hamad Medical Corporation, Doha, Qatar; 2 Department of Internal Medicine - Hematology and Oncology, University Hospital Brno and Masaryk University, Brno, Czechia; 3 Department of Pharmacy, Heart Hospital, Hamad Medical Corporation, Doha, Qatar; 4 Department of Pharmacy, National Center for Cancer Care and Research, Hamad Medical Corporation, Doha, Qatar; 5 Division of Hematology, Department of Internal Medicine, Cerrahpaşa Faculty of Medicine, Istanbul University-Cerrahpaşa, Istanbul, Türkiye; 6 Department of Clinical and Biological Sciences, University of Turin, Turin, Italy; 7 Department of Hematology, National Center for Cancer Care and Research, Hamad Medical Corporation, Doha, Qatar; 8 College of Medicine, Qatar University, Doha, Qatar

**Keywords:** anesthesia, chronic myeloid leukemia, hematology, perioperative management, tyrosine kinase inhibitors

## Abstract

**Background/objectives:**

Patients with chronic myeloid leukemia (CML) are increasingly undergoing surgical procedures as long-term survival improves with tyrosine kinase inhibitor (TKI) therapy. This narrative review aims to synthesize current evidence regarding perioperative risk assessment and management in patients with CML receiving TKIs.

**Methods:**

A literature search was conducted using structured searches of PubMed, Scopus, Web of Science, and Google Scholar for studies addressing perioperative management, anesthesia, bleeding, thrombosis, wound healing, obesity, pregnancy, and drug interactions in CML. Original studies, retrospective cohorts, case series, case reports, and narrative reviews relevant to perioperative care were included.

**Results:**

Available evidence demonstrated that perioperative risk in CML extends beyond routine hematologic parameters and is strongly influenced by TKI-specific toxicities and disease-related factors. Dasatinib showed the greatest association with platelet dysfunction and cytopenias, while nilotinib and ponatinib were linked to increased arterial and cardiovascular risk. Splenomegaly, obesity, bariatric surgery, and pregnancy further altered perioperative risk and TKI pharmacokinetics. Available evidence does not establish validated drug-specific preoperative TKI interruption intervals, and the decision regarding temporary interruption should therefore be individualized according to TKI-specific toxicity, disease status, and procedural risk. The review additionally identified clinically significant CYP3A4-mediated interactions with perioperative anesthetic, antimicrobial, and analgesic agents, suggesting preferential use of propofol-based induction and non-CYP-dependent opioids when feasible.

**Conclusions:**

Perioperative management in CML requires individualized multidisciplinary planning integrating hematologic, anesthetic, surgical, and pharmacologic considerations to optimize surgical safety while maintaining disease control.

## 1 Introduction

Chronic myeloid leukemia (CML) is a myeloproliferative neoplasm (MPN) affecting myeloid, erythroid, megakaryocytic, and lymphoid lineages, with an annual incidence of approximately 2 per 100,000 individuals and nearly 9,000 new diagnoses per year in the United States (1). It is characterized by a reciprocal translocation between the breakpoint cluster region (BCR) gene on chromosome 22 and the Abelson murine leukemia viral oncogene homolog 1 (ABL1) gene on chromosome 9, t(9;22), forming the Philadelphia chromosome (2). The resulting BCR::ABL1 fusion protein is a constitutively active tyrosine kinase that activates downstream pathways including RAS/RAF/MAPK, PI3K/AKT/mTOR, and JAK/STAT, promoting proliferation, inhibiting apoptosis, and expanding leukemic stem cells (3, 4). Clinically, CML may progress from chronic phase to advanced disease. For consistency, this review applies the 2022 International Consensus Classification (ICC), which retains accelerated phase, defined by 10-19% blasts in peripheral blood or bone marrow, peripheral blood basophils ≥20%, or qualifying additional clonal cytogenetic abnormalities, and defines blast phase by ≥20% blasts in peripheral blood or bone marrow or extramedullary blast proliferation (5, 6). The 2022 WHO classification instead recognizes CML as a biphasic disease without a separate accelerated phase; this distinction is acknowledged in the 2025 ELN recommendations (5, 7). With currently available treatments, progression to advanced-phase disease has become uncommon.

The introduction of tyrosine kinase inhibitors (TKIs) has transformed CML from a fatal malignancy into a chronic condition with near-normal life expectancy for many patients. Consequently, increasing numbers of patients with CML undergo elective and urgent surgical procedures. Data from 354 surgeries in MPN cohorts demonstrate that 22% were major and 23% urgent, with persistent risks of perioperative bleeding and thrombosis despite targeted therapy (8). In addition, procedures such as bariatric surgery may alter TKI absorption and pharmacokinetics, complicating perioperative planning (9).

Disease biology and TKI therapy introduce unique perioperative considerations. At the time of diagnosis, splenomegaly and hypersplenism, driven by extramedullary hematopoiesis and splenic sequestration, contribute to anemia, leukopenia, and thrombocytopenia (10). Although massive splenomegaly requiring splenectomy has become uncommon, these patients remain at increased perioperative risk. Massively enlarged spleens are associated with higher intraoperative blood loss and transfusion requirements (11).

TKIs exert heterogeneous effects on platelet function and endothelial integrity. Dasatinib is most consistently associated with platelet dysfunction, with impaired aggregation reported in up to 85% of patients (12), whereas bosutinib may impair platelet function without clear clinical bleeding. Nilotinib generally preserves platelet function, while ponatinib may inhibit platelet activation despite its association with arterial thrombosis. Asciminib, a first-in-class allosteric STAMP inhibitor, differs mechanistically by targeting the myristoyl pocket and reducing off-target kinase inhibition (13). Experimental studies demonstrate that dasatinib, nilotinib, and ponatinib impair endothelial wound healing and promote endothelial dysfunction with increased expression of adhesion molecules, including ICAM-1 and VCAM-1 (14–16). Dasatinib additionally disrupts vascular endothelial cadherin, increasing microvascular permeability and contributing to pleural effusions (14).

Systemic toxicities further influence perioperative management. TKIs are metabolized through cytochrome P450 pathways and may cause hepatotoxicity requiring interruption (17). QT interval prolongation has been reported in cohort analyses of cancer patients receiving TKIs, occurring in approximately one-third of treatment episodes, particularly with dasatinib and nilotinib, and occasionally leading to serious arrhythmias (18). Patients with chronic kidney disease appear overrepresented among those who develop arrhythmic events, suggesting renal dysfunction amplifies cardiac risk (18).

Despite hematologic control in chronic-phase disease, surgical cohorts of patients with MPN continue to demonstrate elevated rates of perioperative bleeding and thrombosis (8). Laboratory studies confirm that TKIs variably affect platelet signaling and endothelial repair, resulting in heterogeneity of hemostatic profiles across agents (19). These disease- and therapy-specific alterations are not addressed in conventional perioperative guidelines.

As long-term TKI therapy enables near-normal life expectancy, the need for structured, multidisciplinary perioperative planning becomes increasingly important. This review synthesizes available clinical evidence and mechanistic data to provide a practical framework for perioperative management in patients with CML receiving TKI therapy.

## 2 Methods

This manuscript was conducted as an expert narrative review and was not restricted to a predefined publication period and was designed to provide a comprehensive, practice-oriented synthesis of perioperative considerations for patients with CML. The literature search was guided by the following focused clinical questions:

(1) What disease- and treatment-related factors influence the perioperative risk in patients with CML? (2) How do TKIs affect perioperative hemostasis, cardiovascular risk, wound healing, and drug interactions? (3) What perioperative strategies can optimize surgical outcomes while maintaining disease control in patients with CML? To enhance clinical applicability, the findings are presented according to the chronological phases of perioperative care, including preoperative evaluation, intraoperative management, postoperative management, and special considerations.

Relevant studies were identified through structured keyword searches of PubMed, Scopus, Web of Science, and Google Scholar. Search terms included combinations of “chronic myeloid leukemia” or “CML,” “tyrosine kinase inhibitors,” “TKI,” “perioperative,” “surgery,” “anesthesia,” “bleeding,” “thrombosis,” “splenomegaly,” “obesity,” “pregnancy,” and “drug interactions.” In addition, targeted searches were performed for individual TKIs and their pharmacologic, hematologic, and vascular effects.

Eligible studies included original research articles, retrospective cohort studies, case series, case reports, and narrative reviews that provided data relevant to perioperative risk assessment or management in patients with CML. Only articles published in English were included. Studies were selected based on their relevance to the predefined clinical questions, methodological quality where applicable, and clinical applicability, rather than study design alone, given the limited availability of high-level perioperative evidence in this population. As this was an expert narrative review, the objective was to provide a comprehensive and clinically focused synthesis of the available literature. Accordingly, no formal study screening process, standardized data extraction, or formal quality assessment was undertaken. Consistent with the methodology of an expert narrative review, the included evidence was instead critically appraised through a narrative synthesis of the methodological strengths, limitations, and clinical applicability of the available literature.

## 3 Preoperative evaluation and optimization

### 3.1 Goals of preoperative evaluation

In the perioperative setting, the goals of patient evaluation are derived from the specific risk profiles imposed by the underlying CML pathology and concurrent TKI therapy. Several studies have shown that TKIs can cause measurable alterations in platelet aggregation; however, these laboratory changes often do not correlate with bleeding events. Accordingly, laboratory evidence of TKI-associated platelet dysfunction should not be interpreted as equivalent to an increased clinical bleeding risk (20). Thus, the patient’s treatment history and disease phase may be more informative than isolated laboratory tests alone (20). The contemporary CML population concomitantly presents with other comorbidities, predominantly obesity and its associated metabolic effects. These features can alter the absorption and clearance of TKIs and may add a cardiovascular burden perioperatively (9). Taken together, these factors direct attention to understanding comorbidities, organ function, and hematologic patterns to gauge the risk of bleeding, thrombotic events, or infection in the perioperative period.

### 3.2 Disease status and molecular response

Prior to surgery, the patient’s current disease status should be established, as it directly influences the safety and timing of operative intervention (5). This assessment should include determination of the disease phase (chronic, accelerated, or blast phase) together with evaluation of treatment response using hematologic and molecular response milestones, including BCR::ABL1 transcript levels on the International Scale (IS), which is a standardized reporting system that allows transcription levels to be compared consistently across laboratories and over time (1, 5). Patients with newly diagnosed or uncontrolled disease may require further disease-directed treatment before elective surgery, whereas those with stable chronic-phase disease and sustained treatment responses are generally better candidates for planned operative procedures. In contrast, accelerated and blast phase CML are associated with inferior treatment responses and poorer survival outcomes and frequently require escalation of therapy, including second- or later-generation TKIs, combination chemotherapy in blast phase, and consideration of allogeneic hematopoietic stem cell transplantation, which may preclude or delay elective surgery (21).

Treatment response follows a hierarchical sequence, beginning with complete hematologic response (CHR), followed by cytogenetic response and ultimately molecular response, with each milestone reflecting progressive reduction in leukemic burden (22). While CHR is characterized by normalization of peripheral blood counts and resolution of disease-related clinical features, molecular response is quantified according to BCR::ABL1 transcript levels, with major molecular response (MMR) and deep molecular response (DMR) representing increasingly lower levels of residual disease (5, 22). Achievement and maintenance of these response milestones are associated with improved progression-free and overall survival and have become key therapeutic goals in contemporary CML management (1, 5). Additionally, patients in established treatment-free remission (TFR) represent a distinct perioperative population. Unlike patients receiving active TKI therapy, individuals in established TFR are not exposed to TKI-related perioperative considerations such as drug-drug interactions, platelet dysfunction, endothelial effects, or decisions regarding perioperative TKI interruption. Therefore, establishing whether a patient remains on active TKI therapy or has achieved stable TFR forms an important component of the preoperative assessment (5). Assessment of disease phase, molecular response, and current TKI status, including whether the patient is receiving active therapy or is in established TFR, forms an essential component of the preoperative evaluation and informs subsequent perioperative management.

### 3.3 Hematologic assessment

Patterns of hematologic toxicity observed across TKI clinical trials show agent-specific variation in the severity of TKI-induced cytopenias. Severe neutropenia and thrombocytopenia occur most often with dasatinib, reaching 20–35%, while imatinib is associated with lower rates, and nilotinib with the lowest high-grade event rates (23). These hematologic disturbances in blood cell counts may be transient; as early drops resolve within the first three months of therapy, suggesting a reversible suppression rather than sustained bone marrow failure (24). Given that the available evidence is primarily derived from chronic-phase CML, a phase characterized by bone marrow stabilization with treatment, the resulting patterns are specific to that phase and may not generalize to other phases. Reports describing patients in CML phases other than the chronic phase reported more pronounced cytopenia, with limited perioperative detail (23). These patterns suggest that preoperative evaluation is best informed by current hematologic and organ function parameters, with particular attention to patients receiving dasatinib, which is the TKI most frequently associated with cytopenias.

### 3.4 Hemostasis, thrombosis, and splenic considerations

Hemostatic changes in CML often reflect the combined effect of rising blood cell counts, endothelial stress, and altered coagulation activity, particularly during the first weeks to months after diagnosis when leukocytosis and thrombocytosis are most pronounced. Studies in hematologic malignancies show that thrombin generation and platelet activation tend to occur in the setting of leukocytosis and thrombocytosis, while natural anticoagulant pathways become less effective, creating a state of co-existing increased thrombotic potential and periods of hemostatic instability (25). These procoagulant disturbances are more noticeable in the context of leukocytosis or marked thrombocytosis, which can increase the viscosity of the circulating blood and impair microvascular flow, contributing to disseminated coagulation abnormalities that characterize the leukemic states (26). Similar patterns are reported within the spleen. Increased blood cell counts can cause fluctuations in perfusion within the splenic microcirculation, with splenic infarction reported in this setting. This suggests that the spleen becomes an early site where vascular stress becomes clinically evident (27).

In patients with massive splenomegaly, surgical reports describe increased vascular friability, greater blood loss, and more technical difficulty due to the enlarged hilum and congested splenic tissue. This reinforces the need to identify splenic involvement during the preoperative assessment using imaging such as abdominal computed tomography (CT) or ultrasonography (28).

On the other hand, progressive enlargement of the spleen can culminate in hypersplenism, which is characterized by sequestration leading to lower circulating platelets or red blood cells. These reductions compromise the patient’s hematologic reserve before surgery. Overall, changes in coagulation profiles, platelet indices, and splenic size give practical indicators for CML patient stratification along the preoperative thrombosis-bleeding risk spectrum.

### 3.5 Infection and immunocompromise

Although uncommon in the era of effective TKI therapy, infection risk increases when splenic function is impaired, either from progressive splenomegaly, infarction, or prior splenectomy, all of which reduce clearance of encapsulated bacteria and weaken adaptive responses (29, 30). Preoperative review should therefore verify vaccination status, since pneumococcal, meningococcal, and *Haemophilus influenzae* type b vaccines lower the risk of severe post-splenectomy infection and are most effective when administered at least two weeks before elective surgery (31).

### 3.6 Other considerations

Beyond hemostatic and splenic changes, comorbid organ disease and TKI choice also shape the perioperative risk. Population data show that cardiovascular risk factors are frequent at CML diagnosis, and arterial events occur more often with nilotinib and ponatinib than with imatinib, particularly in patients who already have a medical history of hypertension, dyslipidemia, or coronary artery disease (32–34). Nilotinib is additionally associated with increased rates of new-onset diabetes and hyperlipidemia compared to other TKI agents (33–35). Preoperative optimization should therefore recognize patients receiving nilotinib or ponatinib as being at high cardiovascular risk. Statin therapy may be considered, blood pressure and glucose levels should be strictly controlled, and cardio-oncology input should be sought where available (32, 33). For renal or hepatic dysfunction, standard-dose imatinib, dasatinib, or nilotinib can be used with close monitoring, planned interruptions, and dose reduction when glomerular filtration or liver tests worsen (32).

### 3.7 Therapy review of tyrosine kinase inhibitors

The preoperative impact of TKIs becomes clearer when their pharmacologic differences are viewed in terms of their effects on organ systems relevant to anesthesia. Imatinib, through off-target inhibition of c-KIT and platelet-derived growth factor receptor (PDGFR), can lead to fluid retention and episodes of hepatic dysfunction. These effects may require avoiding excessive perioperative intravenous (IV) fluids and using reduced fluid volumes to limit TKI-associated fluid retention and may also affect drug clearance before surgery (36). Dasatinib’s broader suppression of SRC-family kinases at least partially explains its association with pleural effusions and the relatively complication of pulmonary arterial hypertension. Both complications may compromise respiratory reserve and complicate anesthetic induction or positive-pressure ventilation. Despite its targeted action, nilotinib has been linked to arterial events, hyperglycemia, and hyperbilirubinemia, emphasizing the need to evaluate cardiovascular and metabolic status during the preoperative stage (17). Bosutinib is often associated with diarrhea and hepatotoxicity, creating concerns about hydration status and liver function. In contrast, ponatinib is associated with a high rate of arterial occlusive events (37). More recently, asciminib was initially established in the later-line setting for patients with chronic-phase CML previously treated with multiple TKIs, as demonstrated in the ASCEMBL trial (38). More recently, frontline efficacy has been demonstrated in newly diagnosed chronic-phase CML in the phase 3 ASC4FIRST trial (39), in which asciminib achieved higher MMR rates at 48 weeks than investigator-selected TKIs (67.7% vs 49.0%). These findings distinguish its later-line evidence base from its emerging frontline role.

Given their reported effects on hemostasis, angiogenesis, and endothelial integrity, temporary TKI interruption is frequently undertaken prior to elective surgery because of concerns regarding impaired wound healing and perioperative complications (40). However, evidence supporting this practice is derived from predominantly mechanistic and preclinical studies without comparative perioperative data. There is no standardized evidence-based perioperative guidelines defining drug-specific interruption intervals for these agents. In clinical practice, preoperative interruption is therefore inferred from pharmacokinetic principles, particularly the terminal elimination half-life (t½), which reflects the final phase of systemic drug clearance (41). After approximately 4–5 terminal half-lives, more than 94–97% of a drug is expected to have been eliminated from the systemic circulation. Importantly, pharmacokinetic elimination cannot be assumed to correspond to recovery of pharmacodynamic effects relevant to surgery, including platelet function, endothelial integrity, angiogenesis, or wound healing. We therefore calculated five-terminal-half-life values for each agent as theoretical pharmacokinetic clearance estimates based on published pharmacokinetic data, rather than as clinically validated preoperative interruption periods (41).

Reported terminal elimination half-lives are approximately 18–20 hours for imatinib, 3–5 hours for dasatinib, 17 hours for nilotinib, 32–41 hours for bosutinib, and 27.4 hours for ponatinib (36, 42). For asciminib, the apparent terminal half-life ranges from 7–15 hours (13). Using the upper bound of each reported half-life to ensure conservative estimation, five half-life calculations yield the following approximate clearance intervals: dasatinib (5 h × 5 ≈ 25 hours) corresponding to 1–2 days; asciminib (15 h × 5 ≈ 75 hours) corresponding to 3–4 days; nilotinib (17 h × 5 ≈ 85 hours) corresponding to approximately 4 days; imatinib (20 h × 5 ≈ 100 hours) corresponding to 4–5 days; ponatinib (27.4 h × 5 ≈ 137 hours) corresponding to approximately 6 days; and bosutinib (41 h × 5 ≈ 205 hours) corresponding to 8–9 days. Given the established arterial occlusive and vascular risk profile associated with ponatinib and, to a lesser extent, bosutinib, a more conservative 7–10 day interruption window may be considered for high-risk surgical procedures. These intervals represent theoretical pharmacokinetic models derived from terminal elimination half-lives and reflect expert opinion rather than clinical trial or perioperative outcome data. Consequently, they should not be interpreted as evidence-based recommendations and should instead be individualized according to procedural bleeding risk, cytopenias, cardiovascular comorbidity, multidisciplinary clinical judgment, and overall patient stability. The adverse effects, perioperative considerations, and theoretical five-terminal-half-life systemic-clearance estimates for each TKI are summarized in **Table 1**.

**Table 1:** Adverse effects and clinical implications of TKIs in CML.

Agent	Major adverse effects	Preoperative implication	Suggested preoperative approach	Suggested perioperative interruption interval^†^
Imatinib	Fluid retention and hepatotoxicity (36)	Edema or reduced hepatic clearance	Hold if there is significant hepatic dysfunction	4–5 days preoperatively
Dasatinib	QT prolongation, pleural effusion, and pulmonary hypertension (36)	Lower respiratory reserve	Hold if pleural effusion is present and assess the respiratory status	1–2 days preoperatively
Nilotinib	Arterial events, QT prolongation, hyperglycemia, and hyperbilirubinemia (17)	Cardiovascular and metabolic risk	Cardiac review and optimize cardiometabolic measurements	4 days preoperatively
Bosutinib	Diarrhea and hepatotoxicity (36)	Dehydration or impaired hepatic clearance	Ensure adequate hydration and check liver function tests	8–9 days preoperatively
Ponatinib	High arterial occlusive event rate (17, 37)	Significant arterial thrombotic risk	Hold and assess vascular status	6 days preoperatively
Asciminib	Thrombocytopenia and mild hypertension (13, 38, 39)	Cytopenias, relatively low arterial thrombotic risk	Hold in high-risk surgery or if cytopenia or cardiovascular instability are present	3–4 days preoperatively

^†^Proposed interruption intervals are hypothetical, pharmacokinetically derived estimates based on terminal elimination half-lives and represent expert opinion rather than evidence-based clinical recommendations. They should be individualized according to disease status, bleeding risk, cardiovascular comorbidity, cytopenias, procedural complexity, and multidisciplinary clinical judgment.

Abbreviations: CML, chronic myeloid leukemia; TKI, tyrosine kinase inhibitor.

### 3.8 Preoperative coordination and planning

Preoperative optimization in CML requires the coordination among hematology, anesthesia, and surgical services, particularly when TKI exposure influences cardiovascular or hematologic stability. Case reports show that patients with additional thrombotic risk require individualized perioperative decisions, including adjustments to anticoagulation and TKI timing (43). Elective procedures permit complete evaluation of these issues, whereas urgent surgery prioritizes hemodynamic stability, transfusion readiness, and modification of the anesthetic plan when respiratory and vascular concerns are present. Perioperative management should also account for procedural context, distinguishing minor or low-risk procedures from major or higher-risk surgery when considering TKI interruption and perioperative optimization. Medication reconciliation remains essential, as TKI metabolism varies with obesity, organ dysfunction, or the use of interacting antibiotics and antifungals, making a structured risk review essential, as summarized in **Table 2** (9).

**Table 2:** Preoperative risk factors and optimization strategies in CML patients.

Risk factor	Clinical significance	Optimization strategy
Cytopenias	Lower hematologic reserve	Check CBC, correct cytopenia, and prepare blood products (23)
Thrombotic tendency	Higher arterial risk with some TKIs	Adjust anticoagulants, review TKI choice (26)
Cardiovascular disease	Increased vascular events with nilotinib and ponatinib	Optimize metabolic profile (33)
Renal or hepatic dysfunction	Alter clearance of TKIs and anesthetics	Review organ function, adjust TKI dose or timing (32)
Splenic Dysfunction	Infection susceptibility and sequestration	Confirm vaccines, consider perioperative antibiotics (31)
Drug Interactions	TKIs interact with CYP-metabolized antibiotics and antifungals	Complete medication reconciliation, avoid strong CYP3A4 modulators (9)

CBC, complete blood count; CML, chronic myeloid leukemia; CYP3A4, Cytochrome P450 3A4; TKI(s), tyrosine kinase inhibitor(s).

## 4 Intraoperative management

### 4.1 Intraoperative coordination and hemodynamic management

Coordination among anesthesia, surgery, and hematology services is necessary in CML because several intraoperative complications can occur abruptly, including hemodynamic instability, rapid blood loss, and perfusion changes. In the case of complicated surgical procedures, reliance on non-invasive blood pressure measurements alone may be insufficient to detect rapid hemodynamic changes. Continuous arterial pressure monitoring, active volume assessment, and a clear plan for transfusion timing are therefore essential for detecting and correcting these changes in real time (28).

Patients on TKI therapy who have high thrombotic risk present a different challenge. During procedures requiring adjustment or withholding of anticoagulation therapy, the surgical team should closely monitor for abrupt changes in perfusion, as vascular reactivity and clotting behavior are unpredictable in this setting. This requires shared decisions on maintaining stable blood pressure and enabling prompt response to changes in vascular tone (43). Furthermore, in rare cases with uncontrolled disease or marked leukocytosis, hematologic malignancies predispose patients to hemodynamic instability during anesthesia due to the elevated cell counts and endothelial activation. This suggests early arterial line insertion, temperature control, and continuous communication among team members regarding the evolving hemodynamic targets (25).

### 4.2 Anesthetic drug interactions

Most TKIs are metabolized by Cytochrome P450 3A4 (CYP3A4), and nearly 60% of treatment episodes involve at least one potential interaction with common perioperative medications (44). For anesthetic induction, these pharmacokinetic considerations may suggest the use of agents with minimal CYP3A4 dependence, such as propofol, while minimizing prolonged benzodiazepine exposure when clinically appropriate. Similarly, opioids with limited CYP3A4-mediated metabolism, such as remifentanil or short-acting fentanyl boluses, may be considered to reduce the potential for drug-drug interactions. These considerations are theoretical and are not supported by direct perioperative outcome data in patients with CML. For anesthetic maintenance, volatile anesthetic agents exert their effects on cellular signaling pathways. Isoflurane has been shown in experimental models to induce caspase-3 activation and promote cellular injury signaling (45). In contrast, sevoflurane has demonstrated inhibitory effects on leukemic stem and progenitor cell activity in pre-clinical studies (46). No clinical perioperative studies have demonstrated superior outcomes with sevoflurane or any specific maintenance technique in patients with CML. Accordingly, anesthetic technique should be individualized, with attention to drug interactions and QT-prolonging potential rather than presumed antileukemic effects. Likewise, minimizing the use of agents that significantly prolong the QT interval (e.g. ondansetron, methadone) or strongly inhibit CYP3A4 may be considered in patients receiving nilotinib or other TKIs with established cardiac risk.

### 4.3 Hemostasis, transfusion strategy, and VTE prophylaxis

Bleeding during surgery in patients with CML is influenced by splenic congestion, platelet abnormalities, and the variable coagulation patterns found in the hematologic malignancies. In rare cases requiring splenectomy for massive splenomegaly, substantial blood loss has been reported following the hilar occlusion, making the preemptive availability of packed red blood cells, platelets, and fresh frozen plasma preparations essential to maintain intraoperative stability (28). Since TKI-associated platelet dysfunction does not directly predict surgical hemorrhage, transfusion decisions rely on active bleeding and platelet count trends (20). Intraoperative cell salvage may be considered during high-blood-loss procedures in chronic-phase CML, where circulating blast cells are absent. When intraoperative cell salvage is used, reinfusion through leukocyte depletion filters has not been associated with increased cancer recurrence or mortality in systematic reviews of oncologic surgery populations; however, these data are largely derived from solid tumors, and CML-specific evidence remains limited (47). The British Society for Haematology (BSH) recommends hemostatic support based on laboratory values, including platelet administration during ongoing bleeding and fibrinogen replacement when levels are low (48).

In CML, there is a tendency toward a prothrombotic state under operative stress. This may support the selective use of antifibrinolytics such as tranexamic acid, which should be reserved for cases with bleeding and low thrombotic risk at baseline (25). For venous thromboembolism (VTE) prevention, the International Initiative on Thrombosis and Cancer (ITAC) advises against initiating mechanical VTE prophylaxis as a standalone treatment, and recommends preoperative low-molecular-weight heparin for VTE prophylaxis (48, 49). A recent systematic review suggesting that anticoagulation use concurrently with TKIs in patients with CML appeared feasible, with major bleeding events being uncommon the available literature (50). Key intraoperative risks and corresponding management strategies are summarized in **Table 3**.

**Table 3:** Intraoperative care considerations in CML patients.

Aspect of care	Intraoperative risk	Suggested considerations
Team communication	Sudden hemodynamic instability	Pre-induction briefing and escalation plan (25, 28)
Hemodynamic monitoring	Unstable preload and perfusion, especially with splenomegaly	Early arterial and continuous monitoring (25, 28)
Anesthetic induction	Drug interactions with TKI	Consider agents with limited CYP3A4 interaction; limit benzodiazepines (44)
Anesthetic maintenance	CYP3A4 metabolism and cardiac effects	Sevoflurane or TIVA; avoid QT-prolonging drugs (44, 46)
Blood product preparation	Major intraoperative bleeding	Crossmatch all patients (28)
Antifibrinolytic use	Competing bleeding and thrombosis risk	Use tranexamic acid only with active bleeding and low thrombotic risk (25)
Thrombosis prevention	Perioperative VTE risk	LMWH; mechanical measures as adjuncts (48)

CML, chronic myeloid leukemia; CYP3A4, Cytochrome P450 3A4; LMWH, low molecular weight heparin; TKI, tyrosine kinase inhibitor; TIVA, total intravenous anesthesia; VTE, venous thromboembolism.

## 5 Postoperative management

### 5.1 Immediate postoperative goals

Immediate postoperative management focuses on surveillance for complications and the safe reintroduction of therapy (**Table 4**). In a population-based cohort undergoing cytoreductive nephrectomy, perioperative TKI exposure was independently associated with increased postoperative morbidity at 30 and 90 days, including bleeding events, wound-related complications, cardiovascular events, and systemic medical complications, without an increase in short-term mortality (51).

**Table 4:** Postoperative care considerations in CML patients.

Aspect of care	Postoperative risk	Recommended approach
TKI resumption and hematologic stability	Bleeding, cytopenias, and relapse risk	Resume after hemostasis and clinical recovery (40, 51, 52)
Bleeding and thrombosis	Hematoma, VTE, and arterial events	Monitor bleeding and early ambulation (40, 51–53)
Post-splenectomy state	Infection and thrombocytosis	Vaccinate and consider antibiotics (54–58)
Analgesia and drug interactions	Bleeding and toxicity	Avoid NSAIDs and evaluate CYP interactions (55–57)
Wound healing and infection	Surgical site infection	Wound checks and glycemic control (40)
Cardiac, renal, and discharge planning	Arrhythmia, fluid overload, and non-adherence	Continue cardiac medications and monitor ECG. Adjust dosages and arrange early follow-up (59)

CML, chronic myeloid leukemia; TKI, tyrosine kinase inhibitor; VTE, venous thromboembolism; NSAIDs, non-steroidal anti-inflammatory drugs; CYP, Cytochrome P450; ECG, electrocardiogram.

Accordingly, immediate goals include ongoing monitoring for bleeding, thrombosis, infection, and delayed cytopenias. Given the documented effects of TKIs on platelet activation, endothelial repair, and inflammatory cell recruitment, TKI therapy should be resumed only after achieving hemostasis and early recovery, with continued postoperative surveillance to detect evolving complications (52).

### 5.2 Hematology and therapy resumption

Postoperatively, management transitions from early complication surveillance to optimizing the timely resumption of TKI therapy, while accounting for potential hematologic and vascular risks during early recovery. TKI therapy may be resumed once oral intake is tolerated and adequate hemostasis and wound healing are achieved. The timing of resumption should be individualized according to the type of surgery, wound status, bleeding risk, organ function, and the specific TKI, as available recommendations vary considerably between agents (40, 51). Prolonged interruptions beyond one week should generally be avoided, as extended treatment gaps have not shown to reduce postoperative morbidity and may compromise disease control (51).

Ongoing postoperative care requires structured hematologic follow-up due to the agent-specific effects of TKIs on platelet function, coagulation pathways, and vascular endothelium. These effects may manifest as delayed cytopenias, bleeding complications, or thrombotic events beyond the immediate postoperative window (53). Serial monitoring of hemoglobin, platelet count, and coagulation parameters allows early identification of these complications, particularly during the first postoperative week when late hematoma formation has been described (51). Close follow-up is also relevant after the resumption of TKI therapy, as hematologic toxicity may evolve after re-exposure to therapy.

In patients with postoperative reactive thrombocytosis, platelet counts should be monitored closely, as marked thrombocytosis (1000 × 10^9^/L) has been associated with thrombotic complications. This may support the use of antiplatelet therapy in selected patients (54). In individuals with increased thrombotic risk, including those receiving ponatinib or with a history of VTE, postoperative mobilization strategies and pharmacologic thromboprophylaxis may be incorporated once hemostatic stability permits (53).

### 5.3 Pain control and drug interactions

Postoperative analgesia in patients receiving TKIs should be tailored to hematologic status and pharmacokinetic interactions. Non-steroidal anti-inflammatory drugs (NSAIDs) are generally avoided in the presence of thrombocytopenia or renal impairment, as TKIs are associated with platelet dysfunction and may increase the bleeding risk when combined with agents that inhibit platelet aggregation or reduce renal perfusion (55). Opioid-based analgesia is therefore commonly used, with attention to dose adjustment and monitoring.

These considerations are further influenced by clinically relevant drug-drug interactions in the postoperative setting. Concomitant use of CYP inhibitors, including macrolide antibiotics and azole antifungals, may increase TKI plasma concentrations and toxicity, whereas enzyme inducers may reduce therapeutic exposure (56). Several opioids, including fentanyl, oxycodone, and tramadol, are predominantly metabolized via CYP3A4, the same pathways involved in the metabolism of TKIs, and altered clearance has been associated with increased sedation and adverse effects in cancer patients receiving interacting therapies (57). Therefore, opioids with minimal CYP-mediated metabolism, such as morphine or hydromorphone, are preferred for postoperative analgesia in patients receiving TKIs, while CYP-metabolized opioids (i.e. fentanyl, oxycodone, and methadone) require closer monitoring and dose adjustment regardless of the specific TKI agent used. Accordingly, postoperative pain management should incorporate the review of hepatic metabolism pathways and concurrent medications to reduce the risk of clinically significant drug-drug interactions. NSAIDs should also be avoided to reduce the risk of bleeding or renal injury.

### 5.4 Infection prevention and recovery

Postoperative infection prevention in patients receiving TKIs should be guided by immune risk stratification and procedure type. Patients with functional or anatomical asplenia should receive appropriate vaccination and infection prophylaxis according to current guidelines (58). Patients with treatment-related neutropenia or functional immunosuppression require standard perioperative antimicrobial prophylaxis and early evaluation of febrile illness.

Wound management may be influenced by the anti-angiogenic and endothelial effects of TKIs. Although mechanistic studies suggest delayed tissue repair, the magnitude of clinical wound-healing risk has not been well quantified, and comparative perioperative data remain limited. Attention to local wound monitoring and optimization of perioperative glycemic control is particularly important in patients receiving nilotinib, given its association with metabolic dysregulation (40).

Recovery planning incorporates management of cardiac and renal comorbidities. Continuation of chronic cardiovascular medications, electrocardiogram monitoring in patients receiving QT-prolonging agents, and careful fluid balance are essential during the postoperative period (59). Renal or hepatic dysfunction may necessitate TKI dose adjustment. Early mobilization is recommended to reduce the risk of venous thromboembolism, along with discharge education on medication adherence and early hematology follow-up, typically within 1 to 2 weeks, to reassess treatment tolerance and recovery trajectory.

## 6 Special considerations

### 6.1 Obesity considerations

Following postoperative stabilization and resumption of TKI therapy, obesity introduces additional challenges that warrant targeted planning. Obesity and bariatric surgery can significantly alter the pharmacokinetics of TKIs by impairing absorption and increasing the risk of subtherapeutic exposure or toxicity. Clinicians should anticipate higher risks of wound complications, VTE, and airway difficulty, while also recognizing that standard oral TKI dosing may not achieve therapeutic levels (9). In obese patients undergoing major surgery, closer postoperative hematologic monitoring is warranted compared with non-obese patients, with particular attention to platelet count and hemoglobin trends after the reintroduction of TKIs, given the potential for altered drug absorption and unpredictable hematologic response in obesity.

### 6.2 Pregnancy and obstetric considerations

In obstetric patients, perioperative and peripartum management must balance maternal disease control and fetal safety. Registry data demonstrated that normal childbirth occurred without complications in 76% of pregnancies among women with chronic-phase CML, indicating that favorable obstetric outcomes are achievable with individualized management using interferon-α or delayed TKI use in the later trimesters when clinically necessary (60). Practical treatment algorithms emphasize avoiding TKIs in the first trimester, using interferon for cytoreduction when required, and cautiously introducing imatinib or nilotinib once the 16th week of gestation has been completed if disease control is inadequate (5). Perioperative delivery planning should anticipate cytopenias and bleeding risk; this should be coordinated with a multidisciplinary team.

## 7 Discussion

### 7.1 Evidence summary

This review is among the first to specifically address the perioperative management of patients with underlying CML, a clinical scenario increasingly encountered due to long-term survival with TKI therapy. The primary objective was to distill dispersed clinical evidence and mechanistic data into a practical perioperative approach applicable to surgeons, anesthesiologists, and perioperative physicians.

The available literature is limited and heterogeneous, consisting predominantly of case reports, small case series, retrospective cohort studies not specific to CML, and narrative reviews (**Table 5**) (8, 9, 28, 38, 50, 61–67). Despite this, several perioperative concerns recurred in the literature. These include bleeding risk related to cytopenias and splenic congestion (8, 27), thrombotic complications associated with specific TKIs (33, 34), impaired wound healing (40), and clinically relevant drug-drug interactions (55, 56). While general oncology practice recommends interrupting TKIs for approximately 1–2 weeks prior to elective surgery and resuming therapy only after satisfactory wound healing, that wound healing concern is more relevant with agents that affect angiogenesis and vascular repair, particularly imatinib, dasatinib, and ponatinib. In contrast, newer agents such as asciminib appear to have a more favorable vascular profile (13, 40). Notably, evidence guiding perioperative risk assessment in patients with CML is beginning to evolve beyond case-based reports, with the phase 3 ASCEMBL trial demonstrating improved efficacy and lower treatment-limiting toxicity with asciminib compared with bosutinib (38). Moreover, a recent systematic review synthesizing 13 studies examined the safety of anticoagulation in patients with CML receiving TKIs, including LMWH, vitamin K antagonists, and direct oral anticoagulants, across perioperative and non-perioperative settings. Across the included studies, major bleeding events were infrequent, and severe bleeding was not consistently reported, suggesting that anticoagulation therapy was feasible when clinically required (50). Earlier surgical oncology literature has reported higher rates of bleeding and infection in patients with hematologic malignancies undergoing major surgery, reinforcing modern conclusions that emphasize the need for careful perioperative assessment and individualized management strategies in this patient population (67).

**Table 5:** Summary of clinical evidence on perioperative management in patients with CML.

Study	Number of patients	Disease status	Perioperative context	Conclusion
Gameil (50) (2026) Systematic review	13 studies; no pooled patient number	CML patients receiving TKIs and anticoagulation	Anticoagulation use in perioperative and non-perioperative settings	Concurrent anticoagulation with TKIs was associated with infrequent major bleeding and thrombotic events.
Szuber (8) (2024) Multicenter retrospective cohort	354	MPN, including CML	Major and urgent non-cardiac surgery	Bleeding and thrombosis remained frequent despite the use of TKIs, suggesting individualized perioperative risk assessment.
Haddad (61) (2023) Case-control	22 cases vs 44 controls	CML with prior bariatric surgery	Surgery-TKI interaction	Bariatric surgery was linked to slower molecular response and inferior event-free survival; some improved with dose escalation or switching.
Ghasoub (9) (2025) Narrative review	N/A	Obesity and CML	Bariatric surgery	Obesity and altered anatomy impair TKI absorption, requiring dose adjustments.
Yassin (62) (2021) Case series	5	Chronic-phase CML with obesity	Bariatric surgery (sleeve, banding)	Bariatric surgery caused unpredictable TKI absorption, requiring dose adjustment or agent switching.
Alqatta (28) (2025) Case report	1	Chronic-phase CML with giant splenomegaly	Elective open splenectomy	Giant splenomegaly increased bleeding risk; cytopenia correction and blood preparations enabled safe surgery.
Dahake (63) (2025) Case report	1	Undiagnosed CML	Emergency laparotomy and splenectomy	Spontaneous splenic rupture can be the first presentation of CML; urgent surgery and transfusions were lifesaving
Gonzalez (64) (2024) Case report	1	Post-splenectomy CML	Postoperative hematologic evaluation	Persistent postoperative leukocytosis and thrombocytosis prompted investigation and diagnosis of CML.
Rebahi (65) (2018) Case report	1	Blast-phase CML in pregnancy	Cesarean section	Temporary TKI interruption and general anesthesia allowed safe delivery.
Owsiak (66) (2016) Case report	1	Blast-phase CML in pregnancy	Cesarean section	General anesthesia was preferred over neuraxial techniques due to bleeding risk.
Hohenberger (67) (2005) Narrative review	N/A	Hematologic malignancies, including CML	General surgery	CML patients have higher bleeding and infection risk, requiring correction of cytopenias.

Importantly, mechanistic studies provide a context for these observations. Experimental data demonstrate agent-specific effects of TKIs on platelet signaling, endothelial integrity, and vascular repair, explaining why laboratory platelet dysfunction does not consistently predict surgical bleeding (15, 19, 20, 25). Across the reported surgical cases, bariatric surgery, and obstetric delivery, favorable outcomes were achieved when perioperative decisions were guided by disease phase, hematologic reserve, organ function, and TKI-specific toxicity rather than by the CML diagnosis alone (9, 61). Collectively, these reports suggest a practical, risk-stratified approach that accounts for the patients’ overall clinical status and emphasizes multidisciplinary anticipation for perioperative complications. The evidence synthesized throughout this review has been integrated into a perioperative management framework, which consolidates the key preoperative, intraoperative, postoperative, and special considerations into a practical approach intended to support individualized decision making rather than prescribe rigid management pathways (**Figure 1**).

**Figure 1:**
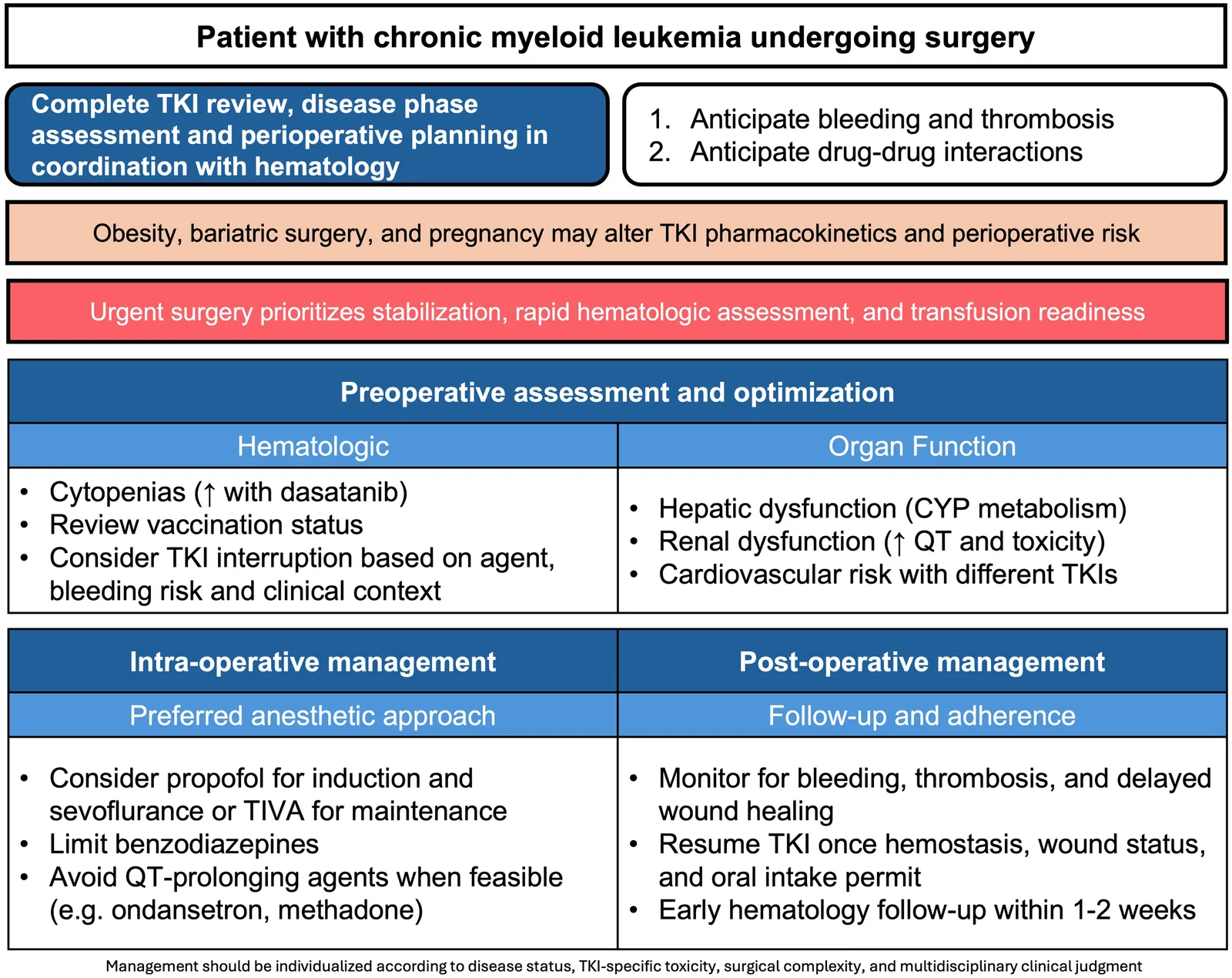
Perioperative care framework in chronic myeloid leukemia.

### 7.2 Critical appraisal and quality of evidence

The evidence informing perioperative care in CML remains limited in both quantity and methodological strength. Gameil et al. provided the highest-level synthesis through a systematic review, but its conclusions regarding concomitant TKI and anticoagulant use were based on a small, heterogeneous literature that included case reports, conference abstracts, and largely non-perioperative settings, limiting reliable estimation of bleeding or thrombotic risk (50). Haddad et al. offered stronger CML-specific comparative evidence through a retrospective propensity-matched analysis of patients with prior bariatric surgery; however, the small exposed cohort, single-center design, limited matching variables, and potential residual confounding prevent delayed molecular response from being attributed solely to impaired TKI absorption (61). The five-patient report by Yassin et al. provides useful clinical observations but remains uncontrolled, non-consecutive, and too heterogeneous to establish the frequency of altered TKI exposure or the effectiveness of dose adjustment or TKI switching (62). Likewise, Ghasoub et al. provide a clinically useful pharmacological synthesis, but the absence of formal study-quality assessment and reliance on indirect bariatric pharmacology and limited CML reports mean that their recommendations remain primarily expert-informed (9).

Direct perioperative evidence is otherwise dominated by isolated cases. Alqatta et al. demonstrated the feasibility of splenectomy after multidisciplinary preparation in one patient, but incomplete reporting of treatment exposure and perioperative outcomes limits assessment of which interventions influenced the favorable result (28). Dahake et al. described conservatively managed spontaneous splenic rupture and therefore contribute diagnostic and non-operative rather than surgical evidence (63). Gonzalez et al. showed that persistent postoperative leukocytosis and thrombocytosis may reveal previously undiagnosed CML, but a single case cannot define diagnostic thresholds or management pathways (64). The obstetric reports by Owsiak et al. and Rebahi et al. describe individualized use of general anesthesia in patients with circulating blasts, yet neither directly compared anesthetic techniques, and the concern regarding neuraxial blast seeding was largely extrapolated from other hematologic malignancies (65, 66). Szuber et al. provide more substantial perioperative data, but their population included polycythemia vera, essential thrombocythemia, and myelofibrosis rather than CML, making its applicability indirect (8). Hohenberger et al. provide useful historical context but predate much of the contemporary TKI era and rely partly on non-CML evidence (67). Collectively, the literature is vulnerable to publication bias favoring unusual or successful cases, selection and survivorship bias within surgically selected populations, residual confounding, and a frequent absence of concurrent controls (8, 9, 28, 50, 61–67). Current recommendations should therefore be interpreted as expert-informed guidance rather than validated perioperative standards.

### 7.3 Research gaps

The current perioperative evidence base in CML is limited and is derived largely from retrospective or heterogeneous hematologic cohorts, which may limit generalizability and blur CML-specific perioperative risks related to disease phase, TKI exposure, and splenic involvement (8, 15, 52). Experience from broader MPN cohorts also shows wide variation in perioperative practice, including frequent interruption of cytoreductive therapy and uncertainty about thromboprophylaxis. Prolonged treatment holds may increase thrombotic risk without clearly reducing bleeding, highlighting the need for shorter, drug-specific interruption strategies in CML rather than blanket discontinuation (8). Rare high-risk scenarios such as massive splenomegaly, splenic infarction, pregnancy, and inherited thrombophilia are described predominantly in case reports, precluding reliable estimation of absolute risk or comparison of alternative management strategies (27, 28, 43).

Mechanistic and pharmacologic studies further expose significant translational gaps. Although TKI-associated platelet dysfunction, endothelial injury, impaired wound healing, QT prolongation, and drug-drug interactions are well documented, these findings have not yet been converted into validated perioperative thresholds or protocols to guide TKI interruption, transfusion support, thromboprophylaxis, or anesthetic planning (18, 19, 40, 55, 56). Likewise, obesity, bariatric surgery, and splenectomy are repeatedly identified as modifiers of TKI pharmacokinetics and hematologic risk, yet robust perioperative outcome data stratified by these factors remain lacking (9, 54). More recently, the evidence base has begun to evolve; however, despite emerging trial data from ASCEMBL, perioperative outcomes and comparative data against higher cardiovascular-risk TKIs remain unavailable, limiting the application of newer agents to perioperative risk stratification (38).

## 8 Conclusion and future directions

The increasing need for surgical care in patients with CML reflects the success of long-term TKI therapy, but it also exposes limitations in traditional perioperative frameworks that were not developed with CML-specific therapies in mind. Perioperative risk in this population is shaped not only by baseline hematologic parameters but also by the interaction between disease phase, treatment-related toxicity, organ reserve, and the physiologic stress of surgery. As a result, uniform drug-holding rules or reliance on isolated laboratory values are insufficient to guide perioperative decision-making.

Across the available literature, a consistent theme emerges: favorable surgical outcomes are most often achieved when perioperative planning is individualized and coordinated across hematology, anesthesia, and surgical teams. Attention to agent-specific toxicity profiles, pharmacologic interactions, and evolving clinical status appears more informative than CML diagnosis alone. Newer therapies, including asciminib, further emphasize this shift, as improved systemic tolerability has not yet translated into clearly defined perioperative risk without dedicated surgical data.

Future progress in this field will depend on moving beyond case-based experience toward structured perioperative data collection. Comparative studies and prospective registries that capture bleeding, thrombotic, and wound-related outcomes across different TKIs, particularly with respect to organ-specific risks, are needed to inform agent-specific perioperative strategies. Until such evidence is available, careful individualized planning, guided by treatment mechanism and patient-specific risk factors, remains the most reliable approach to optimizing perioperative outcomes in patients with CML.
